# 1098. A Phase 1 Safety and Tolerability of Single Ascending Doses of a Novel Engineered Cationic Peptide, PLG0206, in Healthy Subjects

**DOI:** 10.1093/ofid/ofab466.1292

**Published:** 2021-12-04

**Authors:** David Huang, Despina Dobbins, Parviz Ghahramani, Jonathan Steckbeck

**Affiliations:** 1 Peptilogics, Houston, Texas; 2 Inncelerex, Jersey City, New Jersey

## Abstract

**Background:**

PLG0206 is a novel engineered cationic antimicrobial peptide being evaluated for treatment of prosthetic joint infections (PJI). This abstract presents the results from the first in human study to evaluate the safety, tolerability and pharmacokinetic (PK) profile of PLG0206 when administered as an intravenous (IV) infusion.

**Methods:**

6 cohorts of 8 participants were planned to receive escalating single 1-hour IV infusions of PLG0206 at 0.05, 0.125, 0.25, 0.5, 1, 2 and 3 mg/kg dose or placebo. Participants were randomized to receive either PLG0206 (6 per cohort) or placebo (2 per cohort). At each dose level, there were 2 sentinel participants (1 active, 1 placebo) who were dosed at least 48 hours in advance of the other participants in their group. Serial pharmacokinetic samples were taken prior to infusion and up to 48 post infusion. Safety and tolerability was assessed throughout the study. There was at least a 7-day period after dosing at each of the dose levels before dose escalation.

**Results:**

PLG0206 was safe and well tolerated when administered to healthy volunteers at doses ranging from 0.05 and 1 mg/kg. Therapeutic exposures were achieved at 1 mg/kg. The 2 and 3 mg/kg cohorts were not studied. The incidence of treatment emergent adverse events related to study drug administration was low and most events mild (Grade 1) in severity and was similar between the PLG0206 treatment and placebo groups. There were no SAEs, life-threatening events or deaths throughout the study. IV PLG0206 exhibited linear PK over the dose range of 0.05 to 1.0 mg/kg. The median terminal half-life (t½) ranged from 7.37 to 19.97 hours. AUC_0-∞_ increased with increasing PLG0206 dose ranging between 1581.41 and 21141.52 ng.hr/mL. Cmax ranged between 256 and 2653 ng/mL. The mean apparent volume of distribution (Vz) increased was between 25.49 and 94.2 L, mean clearance (CL) were similar across all and ranged from 2.42 to 4.18 L/hour.

**Conclusion:**

Following single IV infusion to healthy volunteers, PLG0206 was safe and well tolerated at doses ranging from 0.05 to 1 mg/kg. IV PLG0206 exhibits linear PK over the dose range. These findings support the ongoing development of IV PLG0206 and will inform dosing regimens in future studies to investigate its utility as an antimicrobial agent.

**Disclosures:**

**David Huang, MD, PhD**, **Peptilogics** (Employee) **Despina Dobbins, BS**, **Peptilogics** (Employee) **Parviz Ghahramani, PhD, PharmD, MSc, MBA**, **Peptilogics** (Consultant) **Jonathan Steckbeck, PhD**, **Peptilogics** (Employee)

